# Wavelet-Detected Changes in Nocturnal Brain Electrical Activity in Patients with Non-Motor Disorders Indicative of Parkinson’s Disease

**DOI:** 10.3390/neurolint16060110

**Published:** 2024-11-16

**Authors:** Anastasiya E. Runnova, Maksim O. Zhuravlev, Anton R. Kiselev, Ruzanna R. Parsamyan, Margarita A. Simonyan, Oxana M. Drapkina

**Affiliations:** 1 National Medical Research Center for Therapy and Preventive Medicine, Moscow 101990, Russia; a.e.runnova@gmail.com (A.E.R.);; 2 Department of Biophysics and Digital Technologies, Saratov State Medical University, Saratov 410012, Russia; 3 Institute of Physics, Saratov State University, Saratov 410012, Russia

**Keywords:** signal processing, oscillational patterns, wavelet analysis, EEG markers, Parkinson’s disease, polysomnography, sleep

## Abstract

Background/Objectives—Parkinson’s disease (PD) is the second most common neurodegenerative disorder caused by the destruction of neurons in the substantia nigra of the brain. Clinical diagnosis of this disease, based on monitoring motor symptoms, often leads to a delayed start of PD therapy and control, where over 60% of dopaminergic nerve cells are damaged in the brain substantia nigra. The search for simple and stable characteristics of EEG recordings is a promising direction in the development of methods for diagnosing PD and methods for diagnosing the preclinical stage of PD development. Methods—42 subjects participated in work, of which 4 female/10 male patients were included in the group of patients with non-motor disorders, belonging to the risk group for developing PD (median age: 62 years, height: 164 cm, weight: 70 kg, pulse: 70, BPsys and BPdia: 143 and 80)/(median age: 68 years, height: 170 cm, weight: 73.9 kg, pulse: 75, BPsys and BPdia: 143 and 82). The first control group of healthy participants included 6 women (median age: 33 years, height: 161 cm, weight: 66 kg, pulse: 80, BPsys and BPdia: 110 and 80)/8 men (median age: 36.3 years, height: 175 cm, weight: 69 kg, pulse: 78, BPsys and BPdia: 120 and 85). The second control group of healthy participants included 8 women (median age: 74 years, height: 164 cm, weight: 70 kg, pulse: 70, BPsys and BPdia: 145 and 82)/6 men (median age: 51 years, height: 172 cm, weight: 72.5 kg, pulse: 74, BPsys and BPdia: 142 and 80). Wavelet oscillatory pattern estimation is performed on patients’ nocturnal sleep recordings without separating them into sleep stages. Results—Amplitude characteristics of oscillatory activity in patients without motor disorders and the prodromal PD stage are significantly reduced both in terms of changes in the number of patterns and in terms of their duration. This pattern is especially pronounced for high-frequency activity, in frequency ranges close to 40 Hz. Conclusions—The success of the analysis of the electrical activity of the brain, performed over the entire duration of the night recording, makes it promising to further use during daytime monitoring the concept of oscillatory wavelet patterns in patients with non-motor disorders, belonging to the risk group for developing PD. The daytime monitoring system can become the basis for developing screening tests to detect neurodegenerative diseases as part of routine medical examinations.

## 1. Introduction

Parkinson’s disease (PD) [1,2] is a disabling neurodegenerative disorder characterized by motor symptoms such as slowness of movement and tremor, as well as non-motor symptoms including cognitive problems such as memory changes, anxiety, and depression and sleep disturbances. Today, in the Russian Federation, this disease has been officially diagnosed in 250,000 to 300,000 patients [3]. In the United States, according to official data from the Parkinson’s Foundation, about one million patients have this diagnosis, and worldwide, about 10 million people suffer from it [4]. Differential diagnosis of this disease is very difficult for doctors and specialists both during the initial diagnosis and during clinical assessment of the patient’s progress. In practice, the modern tool for clinical diagnosis remains the monitoring of motor system abnormalities, although it is undoubtedly very subjective and subject to human error. In [5], it was also reported that the accuracy of clinical diagnosis performed by movement disorder experts is unsatisfactory (the accuracy of the initial assessment is about 80% and the accuracy of the subsequent assessment is 84%). At the same time, by the time motor symptoms appear and, respectively, the diagnosis is made, almost 60–80% of dopaminergic nerve cells are damaged in the substantia nigra of the brain [1]. Thus, the earlier and more accurate detection of PD and, accordingly, initiation of neuroprotective treatment are crucial for improving the prognosis of the disease and, possibly, slowing its progression. Consequently, it is very important to correctly identify risk classes of patients for a quick and clear clinical diagnosis and timely initiation of treatment.

In recent years, several modern deep learning methods have been proposed for PD diagnosis based on electroencephalography (EEG), computer tomography (CT), magnetic resonance imaging (MRI), positron emission tomography (PET), speech tests, and handwriting and sensory data [6]. Shaban [6] states that most deep learning methods use either resting-state EEG or handwriting or sensory data. Compared with CT, PET, and MRI, EEG is a very inexpensive tool used today to diagnose several brain diseases, including epilepsy, tumors, and stroke. In addition, the EEG obtained by polysomnography is the “gold” standard for human sleep research. Today the EEG is easily scalable to high density, can be used at home, and can be easily combined with human motor activity, which makes this method applicable to almost any brain research design.

It is well known that sleep is significantly disturbed in patients with PD. In particular, patients with PD often experience sleep disturbances, including insomnia, rapid eye movement (REM) sleep behavior disorder, and excessive daytime sleepiness [7,8]. In addition, PD is characterized by specific changes in sleep architecture, including a reduction in REM sleep, which may correlate with cognitive impairment associated with its role in the consolidation of procedural memory and motor skills [9]. At the same time, numerous studies in recent years have shown that the sleep microstructure in PD patients is disturbed in both REM and slow-wave, NREM sleep. In particular, it has been shown that during REM sleep, there is an increase in spectral power in the δ (1–4 Hz) and θ (4–8 Hz) ranges, while during NREM sleep stages, on the contrary, the power of σ-waves (12–15 Hz) in the parietal regions decreases [10,11,12,13,14].

However, the approach of sleep hypnogram development, undoubtedly necessary for solving somnology tasks, begins to impose a number of limitations on the researcher when studying the oscillatory structure of EEG activity. Firstly, the algorithms for assessing the hypnogram structure suffer from some arbitrariness and subjectivity associated with their attachment to expert assessment and/or dependence on the selected method of automatic diagnostics [15,16]. In particular, as was shown in the work by Rosenberg and Hout [15], expert assessments can differ from each other by 18–20%. Secondly, the sleep structure can be significantly disrupted in PD patients due to the medications they take, stress, and/or the equipment used in PSG. However, at least some assessments of the sleep microstructure, namely the oscillatory structure of sleep electroencephalography, are quite stable and change little during transitions between sleep stages. In the work by Zhuravlev et al. [17], it is shown that the calculated values of wavelet bicoherence in different frequency ranges retain a very high stability, demonstrating weak deviations in the REM and NREM sleep phases from the average values for the entire duration of sleep. Also, in the work by Runnova et al. [18], for patients with obstructive sleep apnea, they demonstrate the prospects of the approach to assessing the quantitative parameters of oscillatory patterns based on continuous wavelet transformation (CWT) in the EEG for the entire duration of sleep without dividing it into stages for calculating diagnostic markers associated with the underlying disease. It should be noted that in [19], the CWT method was also successfully used as a primary method for processing EEG signals using the classical division of sleep into phases and stages.

In the presented work, we apply CWT to assess oscillatory patterns in polysomnographic recordings of patients with non-motor disorders (NMDs) and healthy volunteers, demonstrating the possibility of identifying disease markers by direct comparison of the entire duration of sleep recordings and their reliable separation in various bands We consider the main group as a group consisting of patients with a high risk of developing PD, since they have a number of non-specific symptoms—anosmia, mild emotional disorder, chronic constipation, etc.—and specific changes in the structures of the brain substantia nigra in MRI and PET. A deterioration in cognitive status, neither by subjective complaints nor by examination data, was not revealed. In addition, close relatives of patients described characteristic changes in night-sleep-talking and frequent night screams, in connection with which the patients were referred for a polysomnographic sleep study procedure. Currently, clinical monitoring of the neurological condition of patients continues.

The mathematical analysis showed a statistically significant change in the structure of the electrical EEG activity of the cerebral cortex, recorded throughout the night, without division into sleep stages. It was shown that these differences are significant when compared with the control group, consisting of both young healthy participants and age-matched practically healthy volunteers.

## 2. Materials and Methods

### 2.1. Materials

**Figure 1:**
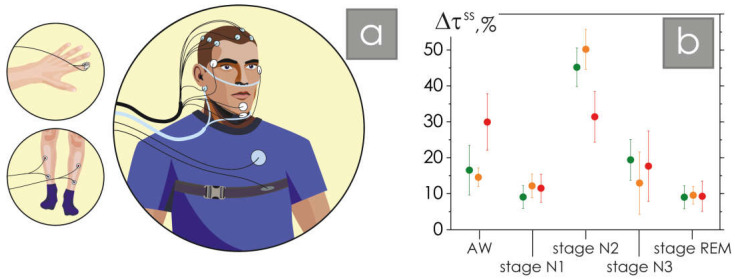
(**a**) Diagram of the arrangement of polysomnographic equipment during a nighttime sleep study of a patient; (**b**) sleep stage duration ∆τ^ss^ distribution diagram: green and orange colors show sleep stage diagrams for groups of healthy young and elderly participants, respectively, and the red color corresponds to the results of sleep assessments in a group of patients with non-motor disorders, where ∆τ^ss^ is the relative duration of the sleep stage to the patient’s total sleep duration, given as a percentage.

Forty-two subjects participated in our work. The study protocol was approved by the Ethics Committee of the National Medical Research Center for Therapy and Preventive Medicine (Moscow, Russia), and all experimental procedures were performed in accordance with the ethical standards laid down in the Declaration of Helsinki. All subjects were informed about the experimental procedures in detail and have signed standard consent forms. The polysomnography, an overnight sleep study recorded for every participant, included registration of the electroencephalography (channels O1, O2, C3, C4), electrocardiogram, photoplethysmogram, respiratory signal, chin and limb myograms, and oculograms, as shown in Figure 1a.

All study participants were divided into three groups—practically healthy volunteers, young and elderly aged, and patients with non-motor disorders (elderly aged). Patients with non-motor disorders suffered from non-specific symptoms: anosmia, mild emotional disorder, chronic constipation, etc. The Montreal Cognitive Assessment (MoCA) test showed no impairment in basic cognitive functions, where all participants received over 28 points. In addition, the patients themselves and their relatives did not complain about the subjective feeling of a decrease in cognitive status. Modern methods of functional neuroimaging—MRI and PET of the brain of patients—demonstrated specific changes in the structures of the substantia nigra in the brain. Close relatives of patients described characteristic changes in night-sleep-talking and frequent night cries. Movement disorders (hypokinesia, tremor, muscle rigidity) were not observed in the study participants. Thus, the group of patients with non-motor disorders can be considered as patients from the high-risk group for the development of PD or the prodromal stage of PD.

A total of 4 female/10 male patients were included in the group of patients with non-motor disorders, belonging to the risk group for developing PD (median age: 62 years, height: 164 cm, weight: 70 kg, pulse: 70, BPsys and BPdia: 143 and 80)/(median age: 68 years, height: 170 cm, weight: 73.9 kg, pulse: 75, BPsys and BPdia: 143 and 82). The first control group of healthy participants included 6 women (median age: 33 years, height: 161 cm, weight: 66 kg, pulse: 80, BPsys and BPdia: 110 and 80)/8 men (median age: 36.3 years, height: 175 cm, weight: 69 kg, pulse: 78, BPsys and BPdia: 120 and 85). The second control group of healthy participants included 8 women (median age: 74 years, height: 164 cm, weight: 70 kg, pulse: 70, BPsys and BPdia: 145 and 82)/6 men (median age: 51 years, height: 172 cm, weight: 72.5 kg, pulse: 74, BPsys and BPdia: 142 and 80).

**Figure 2:**
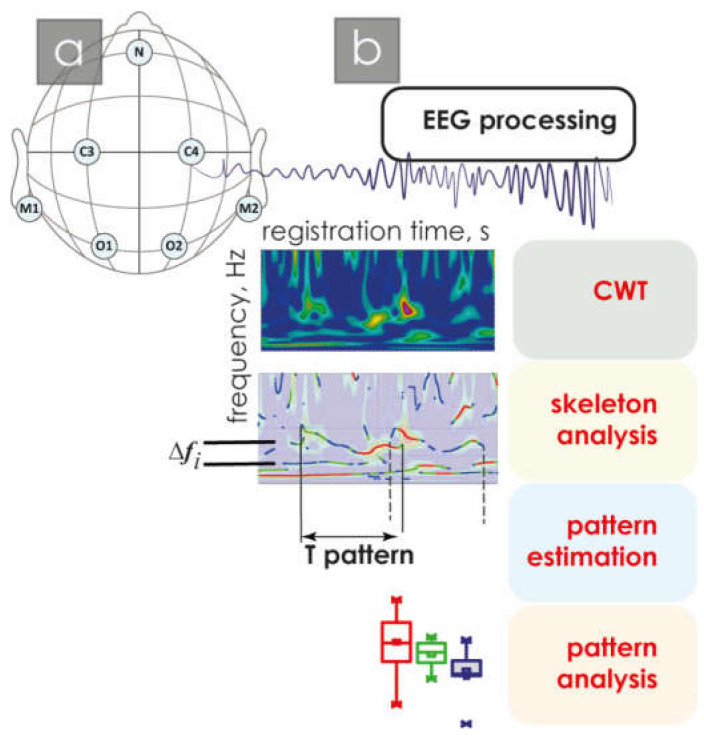
(**a**) Electroencephalography electrode arrangement diagram, where C3, C4, O1, and O2 are active electrodes, and M1, M2, and N are auxiliary electrodes necessary for correct EEG recording; (**b**) scheme of the main stages of EEG signal processing. The bottom panel shows the box diagrams, depicted the following statistical characteristics of third numerical parameters: the first and the quartiles (25–75%, inside the box); the median and the mean (transverse line and point inside the box, respectively); the 1.5 interquartile range (shown by whiskers); and the outliers represented by asterisks.

Standard sleep analysis was performed under the control of an experienced expert sleep specialist. A comparison of the duration of all identified hypnogram stages is shown in Figure 1b. Note that significant differences for the group of patients with NMD and healthy groups were observed only in AW and stage N2. Note that the data in the figure are presented as a percentage, so patients with NMD have a 12–17% decrease in the proportion of slow sleep stage N2 and, at the same time, a 12–17% increase in the proportion of night awakenings. The number of apnea/hypopnea events in all study participants did not exceed 7.6%. For further analysis, electroencephalography signals were used, and the EEG registration is shown in Figure 2a.

### 2.2. Methods

The general scheme of processing each EEG signal is shown in Figure 2b.

#### 2.2.1. CWT—Analysis

The primary stage of signal processing is implemented using a standard continuous wavelet transform [20] of the *EEG(t)* time realization:

(1) $$W\left(s,t_{0}\right)=\frac{1}{\sqrt{s}}\int_{-\infty }^{+\infty }EEG\left(t\right)\psi^{*}\left(\frac{t-t_{0}}{s}\right)dt$$

where $\psi_{s,t_{0}}\left(t\right)$ is the wavelet function obtained from the Morlet mother wavelet with *ω*_0_ = 2*π*. The time scale s determines the wavelet width *ψ*(*s*, *t*_0_ )(*t*), *t*_0_ is the time shift of the wavelet function along the time axis, and the symbol “*” in (1) means complex conjugation. The choice of the wavelet parameter value *ω*_0_ = 2π ensures the relation *s* ≈ 1/*f* between the time scale *s* of the wavelet transform and the frequency *f* of the classical Fourier spectrum.

The wavelet spectrum

(2) $$W\left(s,t_{0}\right)=\left|W\left(s,t_{0}\right)\right|e^{j\varphi_{s}\left(t_{0}\right)}$$

characterizes the behavior of the system on each time scale s at any moment in time *t*_0_. In this case, the value $\left|W\left(s,t_{0}\right)\right|$ characterizes the presence and intensity of the corresponding time scale s at each moment in time t_0.

#### 2.2.2. CWT—Skeleton Analysis

When calculating the wavelet spectrum, a situation of acquiring redundancy of information is realized when, from a one-dimensional EEG signal using the transformation (1), a transition to a two-dimensional plane *W*(*s*,*t*_0_) (2) occurs. In this case, there is an approach to reducing the redundancy of information through the evaluation of skeletons, i.e., maximum components, at each moment of time, as is performed in [18,21,22,23].

In this case, for each moment of time *t*_0_, seven maximum values, $W^{1-7}\left(s_{1-7},t_{0}\right)$, were calculated. In the next moment, seven maximum values, $W^{\prime ,1-7}\left(s_{1-7}^{\prime },t_{1}\right)$, were also estimated. Thus, an array of maximum values of energy components was formed, i.e., the skeleton of the CWT energy surface.

#### 2.2.3. CWT—Pattern Estimation

Further processing allowed us to estimate the oscillatory structures developing in the EEG. According to [22,23], the set of coordinates [$\left(s_{1-7},t_{0}\right),\left(s_{1-7}^{\prime },t_{1}\right),\;\ldots$] was analyzed in the entire EEG time series. The s—the proximity between pairs of coordinates (s, t) at nearby time steps—was estimated. If such a proximity was present, i.e., the distance between a pair of coordinates did not exceed δs = 0.0008, and the two detected extremes were attributed to one oscillatory CWT pattern. The number and duration of patterns were normalized to the average number of patterns per thirty-second time interval.

Next, each pattern was characterized by the following relatively related parameters: duration and fundamental frequency. The fundamental frequency Fm of a pattern is defined as its average frequency Fm≈∑1si, where $s_{i}$ are CWT scales, corresponding to the coordinates of the extreme values defining this pattern. Obviously, the pattern duration is defined as T = T_end_ − T_start_ (see Figure 2b). At the same time, for the main frequency of a pattern, the period can be defined as Tm = 1/Fm. In this case, patterns with duration T shorter than Tm, T < 1/Fm, were considered noise and excluded from further analysis.

All calculations were performed on a high-performance PC equipped with a video card that supports GPU computing technology using CUDA with the following characteristics: CPU: i9-10850K 3.6 GHz; RAM: 64 Gb; GPU: NVIDIA GeForce RTX2070 (8192 Mb).

#### 2.2.4. Statistical Evaluation and Pattern Analysis

For the entire duration of nocturnal sleep, the number N and duration T of oscillatory CWT patterns were estimated in twenty frequency bands: Δ*f*_1_ [1; 2], Δ*f*_2_ [2; 4], Δ*f*_3_ [4; 6], Δ*f*_4_ [6; 8], Δ*f*_5_ [8; 10], Δ*f*_6_ [10; 12], Δ*f*_7_ [12; 14], Δ*f*_8_ [14; 16], Δ*f*_9_ [16; 18], Δ*f*_10_ [18; 20], Δ*f*_11_ [20; 22], Δ*f*_12_ [22; 24], Δ*f*_13_ [24; 26], Δ*f*_14_ [26; 28], Δ*f*_15_ [28; 30], Δ*f*_16_ [30; 32], Δ*f*_17_ [32; 34], Δ*f*_18_ [34; 36], Δ*f*_19_ [36; 38], Δ*f*_20_ [38; 40] Hz.

The mean, median, and standard deviation were used in descriptive statistics of collected data. The Mann–Whitney U test for independent samples was performed for the comparison of quantitative data. The results with a *p* value ≤0.001 were assumed statistically significant. Statistical analyses were conducted by SPSS version 22.0 software for Windows (IBM, Armonk, NY, USA).

## 3. Results

**Figure 3:**
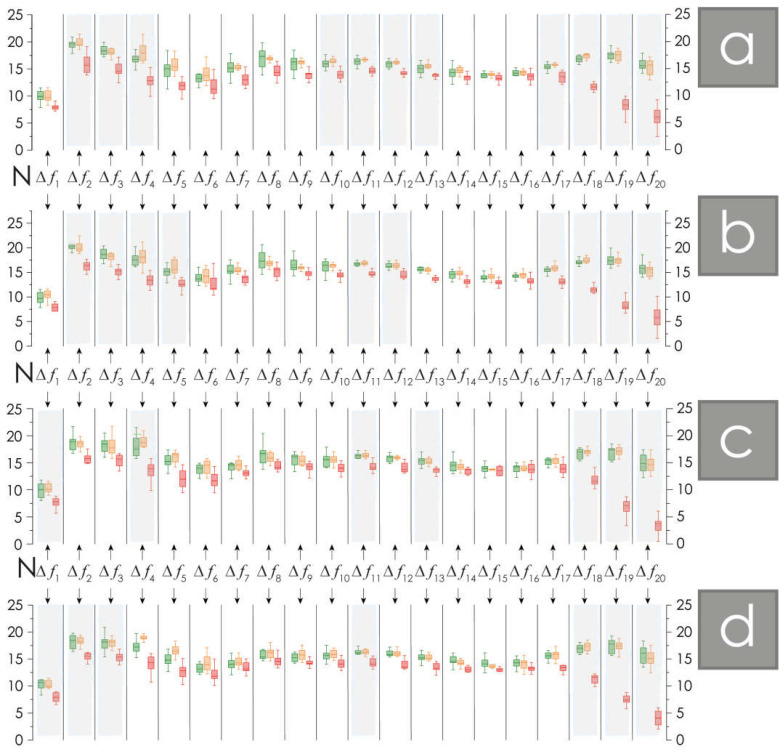
(**a**–**d**) Diagrams of the distribution of the number *N* of patterns in O1, O2, C3, and C4 EEG channels, respectively. The green color corresponds to the data obtained in the group of young healthy participants, the orange color corresponds to the data obtained in the group of elderly healthy participants, and the red color corresponds to the data in the group of patients with non-motor disorders. All diagrams depict the following statistical characteristics of third numerical parameters: the first and the quartiles (25–75%, inside the box); the median and the mean (transverse line and point inside the box, respectively); the 1.5 interquartile range (shown by whiskers); and the outliers represented by asterisks. The diagrams showing statistically significant differences are highlighted in gray, *p* value ≤ 0.001.

The results of the pattern number assessment demonstrate significant differences for the NMD and healthy groups, which are especially pronounced in the high-frequency bands (Figure 3). The number of patterns on the EEG in patients with NMD, a prodrome to eventual Parkinson’s disease, is lower compared to that in the group of healthy volunteers. In the left and right occipital channels O1 and O2, the number of patterns assessed in delta and theta bands, Δ*f*_2_–Δ*f*_4_ [2–8] Hz, significantly differs for patients with NMD compared to healthy participants. The differences revealed are about 20%. Also, in both occipital channels, differences are observed for beta activity, 20–24 Hz, at the level of 15%. The differences in the number of patterns for gamma activity, 32–40 Hz, are maximum and reach more than 60%.

The activity of central channels, C3 and C4, located in the projection of the motor cortex, demonstrate similar dynamics. Low-frequency delta activity in band Δ*f*_1_ [1–2] Hz is characterized by a significant decrease in the number of patterns in patients with NMD (up to 20%). In channel C3, located in the left hemisphere, we also highlight the existence of a reliable decrease in the number of patterns in the following bands Δ*f*_4_ [6–8], Δf11 [20–22], and Δ*f*_13_ [24–26] Hz. These decreases are characterized by a not-too-significant amplitude, namely about 15%. The right-hemisphere channel C4 is characterized by a reliably expressed decrease in the number of patterns in several other bands, namely Δ*f*_2_–Δ*f*_3_ [2–6] and Δ*f*_11_ [20–22] Hz. It should be noted that high-frequency gamma activity, as in the case of the occipital channels, also demonstrates the maximum level of differences. It should be noted that the number of patterns in the band Δ*f*_18_ [34–36] Hz is reduced in patients with NMD compared to healthy volunteers by 31.9%; in the band Δ*f*_19_ [36–38], it is reduced by 60%; and in the band Δ*f*_20_ [38–40], it is reduced by 75.5%.

**Figure 4:**
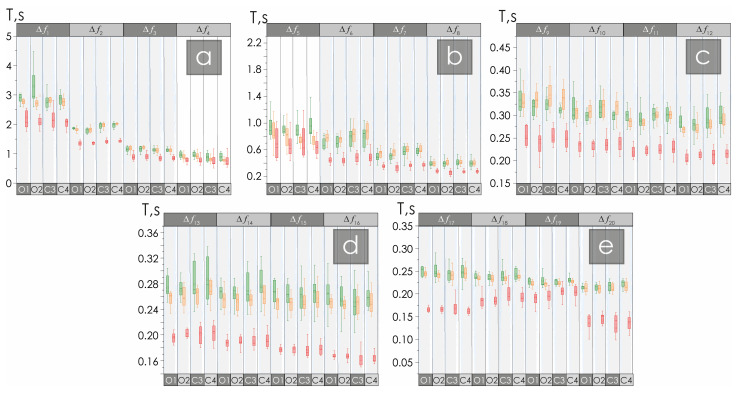
(**a**–**e**) Diagrams of the distribution of the transition T of patterns in Δ*f*_1_–Δ*f*_4_, Δ*f*_5_–Δ*f*_8_, Δ*f*_9_–Δ*f*_12_, Δ*f*_13_–Δ*f*_16_, and Δ*f*_17_–Δ*f*_20_, respectively. The green color corresponds to the data obtained in the group of young healthy participants, the orange color corresponds to the data obtained in the group of elderly healthy participants, and the red color corresponds to the data in the group of patients with non-motor disorders. All diagrams depict the following statistical characteristics of the third numerical parameters: the first and the quartiles (25–75%, inside the box); the median and the mean (transverse line and point inside the box, respectively); the 1.5 interquartile range (shown by whiskers); and the outliers represented by asterisks. The diagrams showing statistically significant differences are highlighted in gray, *p* value ≤ 0.001.

An analysis of the duration of oscillatory CWT patterns is presented in Figure 4. The duration of oscillatory patterns in patients with NMD is significantly reduced compared to healthy study participants by 20–60%. No reliable differences were found for oscillatory frequency bands Δ*f*_4_ and Δ*f*_5_ in all EEG channels, as well as for the C3 channel in bands Δ*f*_18_ and Δ*f*_19_, and for the O2 channel in band Δ*f*_19_.

Age differences between the groups of practically healthy participants are generally not significant, with some exceptions. For example, changes in the number of patterns for the C4 channel in the Δ*f*_4_ and Δ*f*_5_ bands are significant in the groups of young and elderly, practically healthy participants. In addition, the dynamics of the pattern duration for the C3 channel in the Δ*f*_5_ and Δ*f*_13_ bands, for the C4 channel in the Δ*f*_5_ band, and for the O1 channel in the Δ*f*_6_ band change relatively significantly. Despite the described age differences, they do not affect the assessments of the reliability of the features identified in the EEG of night sleep recordings in the NMD patients’ group.

## 4. Discussion

Parkinson’s disease is the second most common neurodegenerative disorder caused by the destruction of neurons in the substantia nigra of the brain. Often, by the time pronounced motor symptoms appear and the correct diagnosis is made, almost 60–80% of dopaminergic nerve cells in the substantia nigra of the brain are damaged [1]. Therefore, clinical diagnosis, still based on the observation of movement disorders, is very limited in its clinical capabilities. Thus, the early diagnosis and timely initiation of effective therapeutic and neuroprotective treatment are crucial to improve the quality of life of patients and potentially slow down the progression of the disease. Although EEG is not currently used for the clinical diagnosis of PD and prodromal stage of PD, many studies have shown its promise in identifying the hallmarks of PD [10,24,25,26,27,28]. Many studies have developed machine and deep learning approaches to differentiate PD from healthy participants based on patients’ staged nocturnal sleep EEG [6,19], using conventional CWT analysis of patients’ EEG as a primary preprocessing step.

In the presented work, we demonstrate the use of the extended CWT-analysis method based on the calculation of oscillatory patterns and their statistical estimates. In the early prodromal period of Parkinson’s disease, the significant oscillatory structure of the electroencephalographic activity of the brain during night sleep is restructuring. Patients without a diagnosis of Parkinson’s disease and without motor disorders already have significant changes in EEG activity, determined by an objective mathematical apparatus. Note that the oscillatory activity in the group of patients with non-motor disorders changes both in terms of the variation in the number of patterns and in terms of their duration. Thus, the structure of oscillatory activity changes both quantitatively and qualitatively. At the same time, an important fact is that significant changes in brain activity in patients with NMD can be identified by averaging the entire total duration of sleep, excluding the procedure for staging night sleep.

At the same time, the analysis of hypnograms of polysomnographic records revealed some decrease in the duration of the N2 slow-sleep stage and a simultaneous increase in night awakenings. However, the relative changes in the duration of these nocturnal processes did not even reach 20%. At the same time, the phase of paradoxical rapid REM sleep did not demonstrate significant changes compared to the control group of healthy participants, which can be a good prognostic sign [12]. Quantitative changes in the characteristics of EEG CWT patterns amounted to more than 20%. Analysis of the changes in high-frequency gamma activity made it possible to see significant variations in EEG characteristics significantly greater than 50%.

The stability of the detected changes in the electrical activity of the brain to age-related features of the brain gives particular importance to the conducted studies. Although age dynamics undoubtedly take place and leave their mark on the dynamics of both the number and duration of patterns [29], the detected changes in brain activity in patients in the prodromal stage of Parkinson’s disease remain pronounced both against the background of young and elderly study participants.

The success of the analysis of the electrical activity of the brain, performed over the entire duration of the night recording, makes it promising to further use the concept of oscillatory CWT patterns in patients with NMD and during daytime monitoring. The daytime monitoring system can become the basis for developing screening tests to detect neurodegenerative diseases as part of routine medical examinations. As in the earlier work by the authors [30], the use of a similar concept of analyzing multichannel EEG data records during nighttime sleep made it possible to identify significant changes in patients with mild cognitive impairment. However, changes in relatively healthy patients were observed in the electrical activity in the frequency band [12; 14] Hz and were not as pronounced in other frequency ranges as observed in this study in patients with NMD, which is interesting in itself, since it may open up the possibility of not only detecting the presence/absence of neurodegenerative diseases, but also determining the severity of the disease in a patient.

However, the prospects for developing screening daytime tests based on oscillatory pattern methods will undoubtedly face the problem of weak statistical data, since, as a rule, routine screening tests within the framework of planned medical examinations rarely exceed 15–20 min, which is almost 15–16 times less than a standard polysomnographic recording. Here, the solution to the problem of poor statistics in this case can be the use of artificial intelligence methods for analyzing the results of assessing oscillatory patterns, close to the methods described, for example, in [19]. Such an expert system can be applied in clinical practice when achieving high efficiency and specificity. Continuing the previously started work [12], subsequent studies will be aimed at identifying changes in the characteristics of oscillatory CWT EEG patterns in neurodegenerative diseases in different stages of sleep and comparing them with the characteristics during the state of calm and active wakefulness.

## 5. Conclusions

This paper examines the oscillatory structure of the electrical activity of the brain of patients with non-motor disorders, in the prodromal stage of Parkinson’s syndrome compared to similar indicators in the control group of healthy participants of different ages. Amplitude characteristics of oscillatory activity in patients with non-motor disorders are significantly reduced both in terms of changes in the number of patterns and in terms of their duration. This pattern is especially pronounced for high-frequency activity, in frequency ranges close to 40 Hz.

## Data Availability

The data presented in this study are available on reasonable request from the corresponding author.
